# Magnetocaloric Effect in an Antidot: The Effect of the Aharonov-Bohm Flux and Antidot Radius

**DOI:** 10.3390/e20110888

**Published:** 2018-11-19

**Authors:** Oscar A. Negrete, Francisco J. Peña, Patricio Vargas

**Affiliations:** 1 Departamento de Física, Universidad Técnica Federico Santa María, Valparaíso 2340000, Chile; 2 Centro para el Desarrollo de la Nanociencia y la Nanotecnología, CEDENNA, Santiago 8320000, Chile

**Keywords:** magnetocaloric effect, quantum dot, Aharonov–Bohm

## Abstract

In this work, we report the magnetocaloric effect (MCE) for an electron interacting with an antidot, under the effect of an Aharonov-Bohm flux (AB-flux) subjected to a parabolic confinement potential. We use the Bogachek and Landman model, which additionally allows the study of quantum dots with Fock-Darwin energy levels for vanishing antidot radius and AB-flux. We find that AB-flux strongly controls the oscillatory behaviour of the MCE, thus acting as a control parameter for the cooling or heating of the magnetocaloric effect. We propose a way to detect AB-flux by measuring temperature differences.

## 1. Introduction

From a fundamental point of view, the magnetocaloric effect (MCE) consists of the temperature variation of a material due to the change of a magnetic field to which it is subjected [1,2,3,4,5,6,7]. Nowadays the research of the MCE effect reawakens a strong interest in the scientific community again [8,9,10,11,12,13,14,15,16,17,18,19,20,21,22,23,24,25,26,27,28,29,30,31,32,33,34,35,36,37,38]. We highlight the works associated with high-temperature caloric materials [21], antiferromagnetic and ferromagnetic interactions [8,15,29,30], heavy lanthanides [31], Fe-Rh alloys [32], among others. In particular, our interest lies in the oscillations of the MCE due the possibility of a wider range of technological applications. In this direction, Reis et al. [33,34,35,36,37,38], describe the oscillations of the magnetocaloric effect in diamagnetic systems (specially in graphene) that can be potentially applied in the construction of magnetic sensors.

**Figure 1:**
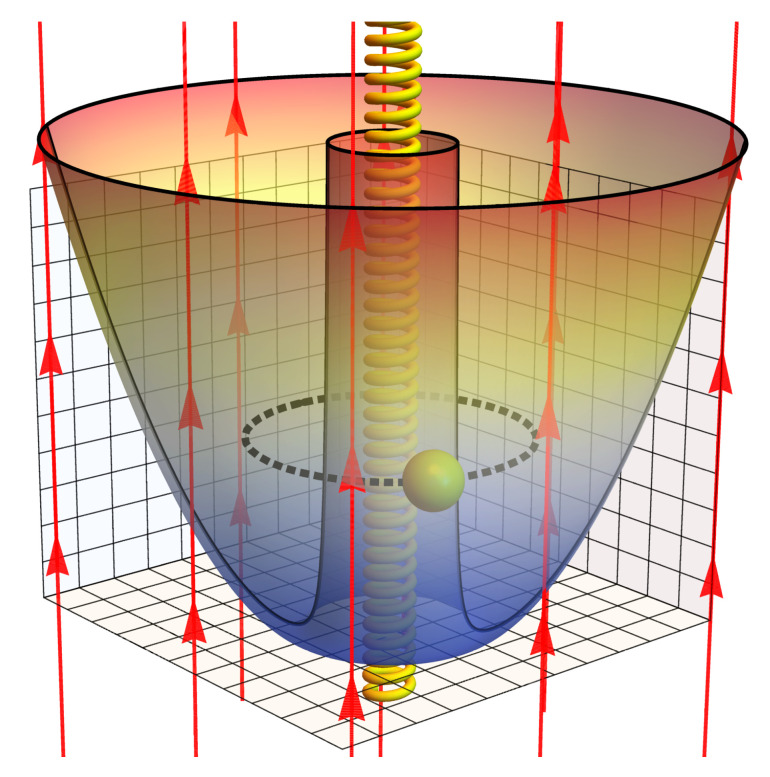
Pictorial representation of an antidot, with an electron trapped in a ring structure subjected to an uniform magnetic field, plus an Aharonov-Bohm flux in the middle of the ring, depicted as an infinite solenoid producing a magnetic field confined inside it.

In physical terms, the MCE is closely linked to the behaviour of the total entropy (*S*) since there is a connection between the temperature changes that a system experiences together with entropy variations. In this context, in a recent work [39], the study of the degeneracy role in the Landau problem shows a very interesting behaviour for the magnetic field along an isoentropic stroke compared with the calculation in his absence. The low-temperature response of the entropy in the Landau problem, only proportional to the amplitude of the external magnetic field, leads to a work where MCE for this problem was being reported, including the case for an electron (with an intrinsic spin) trapped in a quantum dot. Besides, nowadays it is physically possible to confine electrons in two dimensions (2D). For instance, quantum confinement can be achieved in semiconductor heterojunctions, such as GaAs and AlGaAs. At room temperature, the bandgap of GaAs is 1.43 eV while it is 1.79 eV for Al_x_Ga_1−x_ As (x=0.3). Thus, the electrons in GaAs are confined in a 1-D potential well of length *L* in the *z*-direction. Therefore, electrons are trapped in 2D space, where a magnetic field along *z*-axis can be applied [40]. A natural extension of the work [41] corresponds to the study of the magnetocaloric response for an ensemble of antidots. In simple words, an antidot is a potential hill inaccessible to 2D electrons [42,43,44,45,46,47]. The advances in technology allow these systems to work even below T=1 K in temperature [48,49,50,51]. The model used is the one proposed by Bogachek and Landman model [52], that constitutes a combination of repulsive potential ($U\left(r\right)\propto r^{-2}$) and attractive potential ($U\left(r\right)\propto r^{2}$) leaving the electron confined in a finite region of space. Therefore, we investigated a confined electron in a ring topology in the presence of a uniform external magnetic field and subjected to an Aharonov-Bohm flux in the middle of the ring, as shown in Figure 1. In particular, we show that the Aharonov-Bohm flux can be detected by measuring the magnetocaloric effect.

## 2. Model

Let us consider a system given by an electron in the presence of an antidot with an Aharonov-Bohm flux $\left(\Phi_{AB}\right)$ and an external magnetic field B, described by the Bogachek and Landman model. The Hamiltonian which describes the system is given by

(1) $$\begin{array}{c} \hat{H}=\frac{1}{2m^{*}}{\left(p+eA\right)}^{2}+U_{AD}\left(r\right), \end{array}$$

where $m^{*}$ is the effective electron mass, A is the total vector potential, and the term $U_{AD}\left(r\right)$ given by

(2) $$\begin{array}{c} U_{AD}\left(r\right)=\frac{\zeta }{r^{2}}, \end{array}$$

corresponds to a repulsive potential describing the effect of the antidot on the electron. The constant ζ is related to the chemical potential μ and the effective radius of the antidot $r_{0}$ given by the relation

(3) $$\mu =\frac{\zeta }{r_{0}^{2}}.$$

The total vector potential involves two terms, $A=A_{1}+A_{2}$, where $A_{1}$ is related to the external magnetic field $B=\nabla \times A_{1}$, and $A_{2}$ describes the additional magnetic flux $\Phi_{AB}$ inside the antidot. For the case of an external perpendicular magnetic field along the z direction, $B=\hat{z}B$, leads to energy levels for the confined electron

(4) $$\begin{array}{c} E_{nm}=\hbar \omega_{c}\left[n+\frac{{\left[{\left(m+\alpha \right)}^{2}+a^{2}\right]}^{2}+\left(m+\alpha \right)+1}{2}\right], \end{array}$$

where, $\omega_{c}=\frac{eB}{m^{*}}$ is the cyclotron frequency, *n*, *m* are the radial and magnetic quantum numbers and $a^{2}=\frac{2m^{*}\zeta }{\hbar^{2}}=\frac{2m^{*}\mu }{\hbar^{2}}r_{0}^{2}=k_{F}^{2}r_{0}^{2}$, is a constant proportional to antidot radius $\left(r_{0}\right)$, in which $k_{F}$ is the Fermi wave vector of the electron. The values reported for *a* are located in the region of 0≤a≤10 in the original research [52]. The parameter α is defined in the form $\alpha =\frac{\Phi_{AB}}{\Phi_{0}}$, where $\Phi_{0}=\frac{h}{2e}$ is the magnetic flux quantum. Notice that when the parameter α=0 and a=0, the energy levels of Equation (4), take the usual form of the Landau energy levels in cylindrical coordinates. The Landau levels of energy are strongly degenerate for all negative values of *m*, but the inclusion of the antidot repulsive potential in the form of Equation (2), causes the energy levels of Equation (4) to have an asymptotic degeneracy when m→−∞. In addition, we can include a parabolic confining potential $U_{D}$

(5) $$\begin{array}{c} U_{D}\left(r\right)=\frac{1}{2}m^{*}\omega_{0}^{2}r^{2}, \end{array}$$

that modifies the energy levels in Equation (4) as follows

(6) $$\begin{array}{c} E_{nm}^{ad}=\hbar \Omega \left(2n+{\left[{\left(m+\alpha \right)}^{2}+a^{2}\right]}^{1/2}+1\right)+\frac{1}{2}\hbar \omega_{c}\left(m+\alpha \right), \end{array}$$

where $\omega_{0}$ is the parabolic trap frequency, and $\Omega =\omega_{0}{\left(1+{\left(\frac{\omega_{c}}{2\omega_{0}}\right)}^{2}\right)}^{\frac{1}{2}}$. When α=0 and a=0, Equation (6) is reduced to the well-know expression for the Fock-Darwin levels given by

(7) $$\begin{array}{c} E_{nm}^{d}=\hbar \Omega \left(2n+|m|+1\right)+\frac{1}{2}\hbar \omega_{c}m. \end{array}$$

Since a=0 implies ζ=0, we have that the antidot repulsive potential of Equation (2) vanishes and the system then corresponds to a quantum dot.

From here, we can calculate the partition $Z_{ad}$ function, using the general solution of Equation (6), and summing over *n* (n=0,1,2,…) and m=0, ±1,±2,…

(8) $$\begin{array}{c} Z_{ad}=\sum_{n,m}e^{-\beta E_{nm}^{ad}}. \end{array}$$

In particular, when α=0 and a=0, the partition function have an analytical solution given by

(9) $$Z_{d}=\sum_{n,m}e^{-\beta E_{nm}^{d}}=\frac{1}{4}\csc h\left(\frac{\hbar \beta \omega_{+}}{2}\right)\csc h\left(\frac{\hbar \beta \omega_{-}}{2}\right),$$

where the “effective frequency” Ω is defined in the form

(10) $$\Omega =\sqrt{\omega_{d}^{2}+\frac{\omega_{B}^{2}}{4}},$$

and $\omega_{+}$ and $\omega_{-}$ is given by the expression:

(11) $$\omega_{\pm }=\Omega \pm \frac{\omega_{B}}{2}.$$

The complete solution of the MCE for non-interactive quantum dots has been reported for the authors previously, using the analytical thermodynamics from the canonical partition function. Unfortunately, the structure of the energy levels of Equation (6) does not allow a full analytical solution, so we use numerical calculations to obtain the canonical partition function of Equation (8). We separate the contributions of antidot energy $\left(E_{nm}^{ad}\right)$ in the form

(12) $$\begin{array}{ccc} Z & = & \sum_{n}e^{-2\beta \hbar \Omega \left(n+\frac{1}{2}\right)}\sum_{m}e^{-\beta \hbar \Omega {\left[{\left(m+\alpha \right)}^{2}+a^{2}\right]}^{\frac{1}{2}}-\frac{\beta \hbar \omega_{B}}{2}\left(m+\alpha \right)}\\ & = & \frac{1}{2}\mathrm{csch}\left(\beta \hbar \Omega \right)\sum_{m}e^{-\beta \hbar \Omega {\left[{\left(m+\alpha \right)}^{2}+a^{2}\right]}^{\frac{1}{2}}-\frac{\beta \hbar \omega_{B}}{2}\left(m+\alpha \right)} \end{array}$$

**Figure 2:**
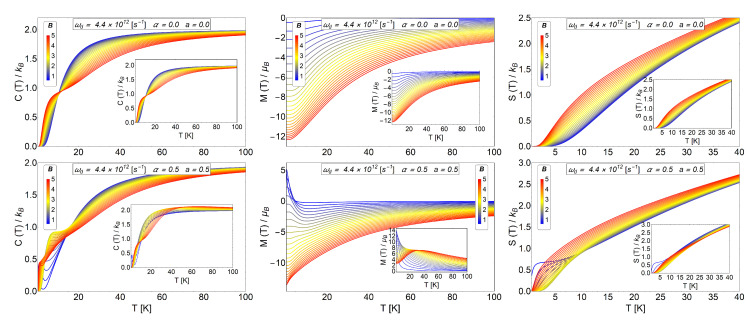
**Upper row:** Specific heat, magnetization and entropy for a quantum dot without intrinsic spin for our numerical calculations using the parameters α=0 and a=0 in Equation (6). The inset images correspond to the exact calculations obtained in the Reference [41] for the same observables. We clearly observe a very good convergence of numerical results. **Lower row:** Specific heat, magnetization and entropy for the case of antidot with a=0.5 and α=0.5. The inset images correspond to the exact calculations obtained in the Reference [41] for the case of an electron in a dot with an intrinsic spin. We observe here similar behaviour at low temperatures for the thermodynamics observables displayed. Therefore, for the thermal observables, the inclusion of AB-flux in an antidot shows similar behaviour as a function of temperature as compared to the case of an electron trapped in a quantum dot with intrinsic spin.

In particular, we work in a range of temperature from 0 K to 100 K that allows us to consider the quantum number m=−300 to m=300 for the energy levels of an anti-dot structure. This selection of values is justified because when we recover the partition function of dot (model of Fock-Darwin), the numerical calculations converge to the analytical results that we display for the specific heat, magnetization and entropy in Figure 2. We can see in the lower row of Figure 2 similar behaviour for the thermodynamics observables displayed for the cases of an electron in a dot with an intrinsic spin and an antidot with the presence of Aharonov-Bohm flux. The MCE effect of the dots with intrinsic spin is fully treated in the Reference [41] and shows that the inclusion of Zeeman term in the formulation produces an oscillatory response of the magnetocaloric observables. Therefore, similar behaviour in the principal thermodynamics quantities for the Bogachek and Landman model is found in our work, making the antidot an interesting candidate for the study of the MCE effect of oscillatory type.

### Magnetocaloric Observables

For the observables ΔT and ΔS we use the following equations

(13) $$\Delta T=-\int_{B_{i}}^{B_{f}}\frac{T}{C_{B}}{\left(\frac{\partial M}{\partial T}\right)}_{B}dB,$$

(14) $$\Delta S=\int_{B_{i}}^{B_{f}}{\left(\frac{\partial M}{\partial T}\right)}_{B}dB,$$

which correspond to standard expressions for the study of the adiabatic change of temperature and the isothermal variation of the entropy in the MCE respectively. For the case of an electron in antidot, we treat two instances, the case with an without Aharonov- Bohm flux (AB-flux). We report that the AB-flux fulfills the same role as the spin term (Zeeman effect) in the MCE reported for quantum dots, that is, the system experiences an MCE of the oscillatory type in the direct-inverse form. It is important to recall that in our thermodynamic analysis, all the thermal quantities are derived from the partition function Z. In the generic form:

(15) $$S\left(T,B\right)=k_{B}T{\left(\frac{\partial \ln Z}{\partial T}\right)}_{B},$$

(16) $$C_{B}={\left(\frac{\partial U}{\partial T}\right)}_{B},$$

where $U=k_{B}T^{2}{\left(\frac{\partial \ln Z}{\partial T}\right)}_{B}$ and finally

(17) $$M=k_{B}T\left(\frac{\partial \ln Z}{\partial B}\right).$$

Before presenting our results, it is essential to clarify that we are using a semi-classical approach to explore the magnetocaloric effect, that is, our adiabatic path corresponds to a process identified in terms of the entropy conservation due to the thermal isolation of the system with the thermal bath [39]. The quantum part is related to the quantum nature of the working substance where the energy spectrum was used to get the classical partition function and used it to analyze the classical adiabatic strokes. We emphasize that the MCE has been studied at systems where considerations like those used in this work reproduce experimental observations in good agreement with the classical theory [53].

## 3. Results and Discussion

The results presented in the next two subsections consider an effective mass $m^{*}∼0.067m_{e}$. This effective mass is associated with a GaAs heterostructures with a typical radius of 20–100 nm [54,55]. For the characteristic frequency of the trap $\omega_{d}$, we use the value of $\omega_{d}=4.4\times 10^{12}$ s${}^{-1}$ which in terms of energy represent approximately $\hbar \omega_{d}∼$ 2.896 meV. The selection of this particular value is in order to compare the intensity of the trap with the typical energy of intra-band optical transition of the quantum dots. The order of this transition is approximately around ∼1 meV for GaAs heterostructure [54]. Finally, in the last subsection, we increase the parabolic trap up to $\hbar \omega_{d}∼5.8$ meV to discuss the effect in the MCE due to changes in $\omega_{d}$.

### 3.1. Influence of Antidot Radius on the MCE

**Figure 3:**
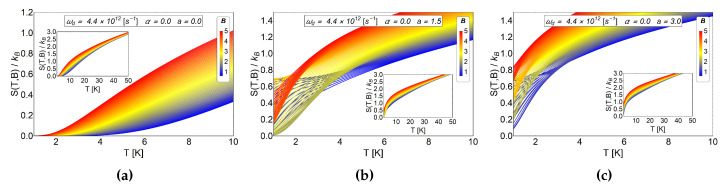
Entropy as a function of temperature for different values of the *a* parameter in absence of AB-flux. The range of the external magnetic field is between 0.6≤B≤5 in Tesla units. (**a**) Entropy for the case of Fock-Darwin energy levels (i.e., α=0,a=0) which represents an electron trapped in a quantum dot. (**b**) Antidot entropy with a=1.5. (**c**) Antidot entropy with a=3.0. We observe in (**b**,**c**), non monotonic behavior of *S* vs *T* for some magnetic fields at low temperatures.

We begin exploring the influence of the antidot radius $r_{0}$ in the thermal response keeping the trap’s frequency at a constant value $\omega_{d}=4.4\times 10^{12}$ $s^{-1}$. To observe only the effect of $r_{0}$, we use α=0 (absence of AB-flux), varying the external field from 0.6 to 5.0 units of Tesla. The Figure 3 shows the entropy as a function of temperature using different values of $r_{0}$ (i.e., the *a* parameter).

**Figure 4:**
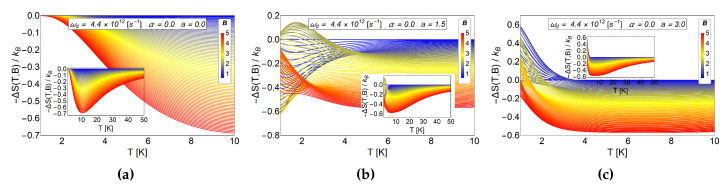
−ΔS as a function of temperatures for different values of *a* parameter in absence of AB-flux. The range of the external magnetic field is between 0.6≤B≤5 in units of Tesla. (**a**)−ΔS for the case of Fock-Darwin energy levels (i.e., α=0,a=0) which represents an electron trapped in a quantum dot. Clearly we always appreciate negative values and absence of crosses for different values of external magnetic field. (**b**) −ΔS for and antidot with a=1.5. (**c**) −ΔS for an antidot with a=3.0. Figure b,c show positive values for −ΔS at low temperatures, T<7 K and then negative values for the entire remaining temperature range.

**Figure 5:**
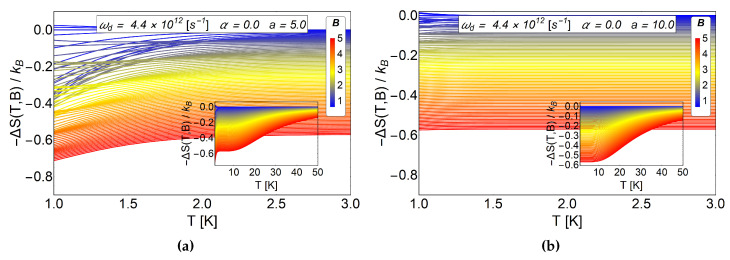
−ΔS as a function of temperature for large sizes of antidot radii. The (**a**) panel correspond to the case of a=5 and the (**b**) panel the case of a=10. Clearly we see that −ΔS<0 therefore direct MCE (ΔT>0) does not occur for this choice of parameters.

For a=0 the entropy grows with the magnetic field, therefore, by calculating −$\Delta S=S\left(T,B_{i}\right)-S\left(T,B_{f}\right)$ with $B_{f}>B_{i}$ we obtain negative values. This result is expected due to the strong degeneracy of the Fock-Darwin levels reflected in the dependence of the spectrum of Equation (7) in the azimuthal quantum number *m*. When *a* starts to increase, the entropy shows an interesting behaviour for low values of *T*, specifically, between 0.1 K to 7 K for (b) panel of Figure 4 also for the values 0.1 K to 3 K for the panel (c) of the same figure. These regions show crosses for low and intermediate values of the external magnetic field and thus giving a way to obtain −$\Delta S=S\left(T,B_{i}\right)-S\left(T,B_{f}\right)>0$ (with $B_{f}>B_{i}$). Therefore, a direct magnetocaloric effect can be obtained in that region. For higher temperatures than those mentioned before, we always found a −ΔS negative and a MCE inverse is recovered. We recall that *a* parameter is associated with $r_{0}$ which can be modified due to experimental set-up. So, this oscillatory type of MCE can be controlled in an experiment. From Figure 4c, we observe that the positive part of −ΔS increases notoriously for a=3, so we expect greater value for ΔT at low working temperature for this set of values. To explore if this effect is enhanced due to an increase in the *a* parameter, we plot −ΔS as a function of *T* for larger values of antidot radii. In Figure 5, we see that the direct MCE effect for a=5 (left panel (a)) and a=10 (right panel (b)) vanishes and we only get −ΔS<0. Therefore, we expect ΔT negative for all temperature region, thus obtaining an inverse MCE solely. To obtain oscillatory behaviour in the MCE, the optimal region of the *a* parameter for the antidot with zero AB-flux, is in the interval 0<a<5.

**Figure 6:**
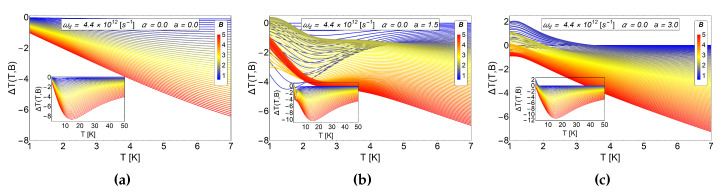
MCE effect for electrons in an antidot in absence of AB-flux. ΔT as a function of temperatures for different values of antidot radii. The (**a**) panel correspond to a values of a=0. The (**b**) panel corresponds to a=1.5 and the (**c**) panel to a=3.0. For all graphics shown here, the initial value of the magnetic field is given by $B_{i}=0.6$ T. The quantity ΔT(T,B) is in units of Kelvin. Here, the horizontal axis represents the initial temperature of the system.

For the MCE observable ΔT, we show the Figure 6 where we appreciate for a magnetic field close to B∼2 T to B∼2.5 T, very small peaks in coherence with the values of −ΔS (lower than 0.1 $k_{B}$) that we can see in the (b) panel of Figure 4. This value of ΔT is more notorious for the case of a=3 reaching a value close to +2 K which is obtained for values close to B=0.7 T. Considering that the initial field is $B_{i}=0.6$ T, we only need a small change in the magnetic field, $\Delta B=B_{f}-B_{i}=0.1$ T, to maximize ΔT at low temperatures.

### 3.2. The Influence of AB-flux in the MCE for Antidots

In this subsection, we treat the case of AB-flux influence in the MCE effect for antidot with different radii. As we discussed in the previous subsection, large antidot radii (a>5) show only an inverse MCE, as well as for small radii (a<1). Therefore, the region of interest is between these two regions for the *a* parameter. The reason for this is because we are looking for an oscillatory response of the MCE with temperature due to AB-flux. So, to quantify and discuss the effect of the AB-flux, we kept the antidot radius at a low constant value. The connection between the α parameter with the AB-flux is given by

(18) $$\begin{array}{c} \alpha =\frac{\Phi_{AB}}{\Phi_{0}}=\frac{AH}{\Phi_{0}}=\frac{\pi r_{s}^{2}H}{\Phi_{0}}, \end{array}$$

where $r_{s}$ corresponds to the radius of the solenoid, H the value of the magnetic field generated by the current inside the same, and $A=\pi r_{s}^{2}$ is the solenoid section area, whose normal vector is parallel to the magnetic field H. We recall that the field H only exists for $0<r\leq r_{s}$ and is zero outside of the solenoid (i.e., for $r>r_{c})$. Thus, for given α, the intensity of the magnetic field inside the solenoid has the form of $H=\alpha \Phi_{0}/\pi r_{s}^{2}$. Recent advances in technology allow fabricating nano-solenoids with a radius of $r_{s}=35$ nm, made by graphene [56]. This result reinforces the idea to explore a small radius for the antidot structure (a<1). Using the value α=0.5, selected for discussions, the value of H is of the order of 0.27 T.

**Figure 7:**
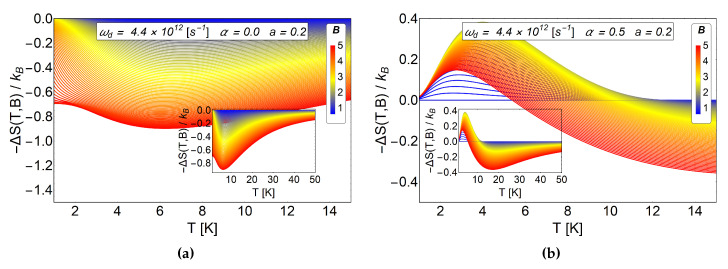
−ΔS as a function of temperature between 0.1 K to 40 K. In the (**a**) panel we consider α=0 and a=0.2 (pure antidot radius effect). In the (**b**) panel we use α=0.5 and a=0.2. We observe notorious positive peak close to 4 K for a direct MCE. The positive peak on the right is caused by the switching on of the AB-flux. For these two graphics, the value of the initial field is $B_{i}=0.6$ T.

First, we plot the value of −ΔS for a small radius of the antidot and a value of α=0.5 fixed in the panel (b) of the Figure 7. We compare those results with those of a=0.2 and α=0 in the (a) panel of the same figure. We see a notorious peak for low temperatures in panel (b) of Figure 7 that can only be associated with the AB-flux, remembering that for small values of the *a* parameter we do not have oscillation in the MCE. Besides, it can be seen that the effect of the AB-flux not only creates an oscillatory direct-inverse magnetocaloric effect but also the inverse response is shifted to higher temperatures, giving the system a wider range of working temperatures.

**Figure 8:**
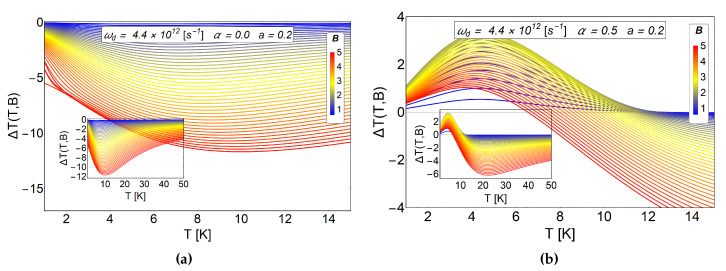
MCE effect for a small antidot radius and the effect of AB-flux. In the (**a**) panel we plot the case in the absence of AB-flux. We observe only the typical inverse response in the MCE effect. The case of α=0.5 is presented in the (**b**) panel, a positive MCE is observed at low temperatures caused by the AB-flux. The quantity ΔT(T,B) is in units of Kelvin. Here, the horizontal axis represents the initial temperature of the system.

The comparison between the ΔT can be appreciated in Figure 8. In the panel (a) we plot ΔT for α=0 and a=0.2 and in the (b) panel we show the results by switching on the AB-flux, maintaining the antidot radius, α=0.5 and a=0.2. We observe a standard inverse MCE in the absence of AB-flux for the small radius of the antidot, as we expected due to the structure that we obtain for −ΔS in the (a) panel of Figure 7. By switching on the AB-flux, α≠0, and using the same radius of the antidot, a positive peak for ΔT∼3.5 K is obtained close to T∼3.5 K for an external magnetic field B∼2.5 T in the case of α=0.5. This peak does not increase as the external field increases, on the contrary, it tends to decrease for magnetic field values larger than $B_{f}>2.5$ T.

**Figure 9:**
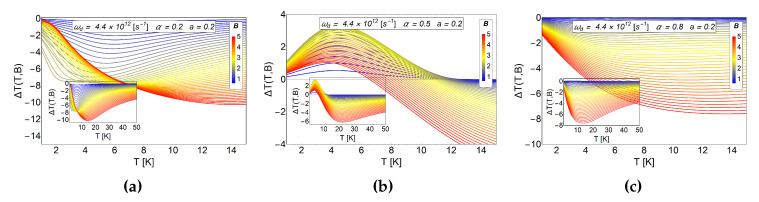
Comparative MCE effect for a fixed small antidot radius and different values of the AB-flux. The *a* parameter is fixed at the value of a=0.2. The (**a**) panel shows the results for the case α=0.2, in the (**b**) panel results for the case α=0.5 and the (**c**) panel, results for α=0.8. The quantity ΔT(T,B) is in units of Kelvin. Here, the horizontal axis represents the initial temperature of the system.

Now we show results for increasing values of the AB-flux. In Figure 9 we show results for ΔT as a function of temperature for fixed value of antidot radius, a=0.2, but different values of α. We observe in the (a) panel of Figure 9 only an inverse MCE effect, even in the presence of AB-flux. The same occurs for α=0.8 as we see in the (c) panel of the same figure. For α=0.5, we appreciated a notorious peak in the (b) panel of Figure 9 as we discussed before. Therefore, increasing α does not necessarily lead to an increase in the positive peak of the MCE. The optimal region for obtaining a MCE of direct type (only associated to AB-flux), is between the values of 0.25<α≤0.5 and low values of *a* (a<1). Outside these values, the two effects (antidot radius and AB-flux) begin to interfere and cannot be differentiated separately.

By reversing the current in the solenoid at the center of the antidot, the AB-flux changes sign, therefore α can be positive or negative. If we consider an AB-flux in the same direction of the applied external magnetic field α has positive values. Opposite case occurs if the flux is contrary to the external field and therefore α takes negative values. This change can be controlled by varying the potential difference applied to the solenoid (i.e., change the direction of the current inside the solenoid).

**Figure 10:**
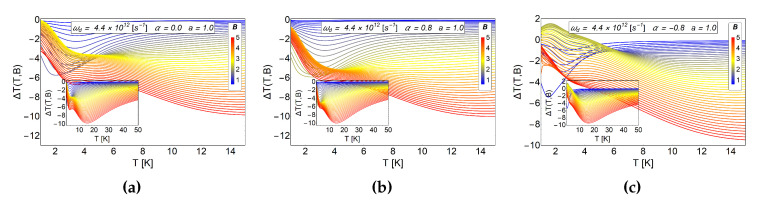
MCE effect for electron in an antidot with AB-flux in different direction. In the (**a**) panel we show the case without AB-flux. The (**b**,**c**) panels shows a comparative MCE effect for a positive AB-flux and negative AB-flux respectively. The quantity ΔT(T,B) is in units of Kelvin. Here, the horizontal axis represents the initial temperature of the system.

In the Figure 10 we observe the two cases previously discussed and the case of α<0. We observe, in the (a) panel and (b) panel of Figure 10, a pure inverse MCE, while in the panel (c) a direct (positive) MCE is obtained. The two first panels (a) and (b), reflect the discussions of the previous subsections. For a<1.5 and in the absence of α we do not expect a positive peak in the MCE, as for the case of α>0.5. The only effect that we expect for α>0.5 is an increased response in the inverse magnetocaloric response as reflected in the panel (b) of the Figure 10, but this is not seen in the entire range of the magnetic field under study. Only the middle values of the external magnetic field (2<B<3 in units of Tesla) they change their value of ΔT notoriously. On the other hand, when α<0, (α=−0.8 for this example) we observe a direct (positive) response in the magnetocaloric effect as we can appreciate in the (c) panel of Figure 10. This interesting response can only be associated with the change in the AB-flux over the sample.

### 3.3. The Role of the Harmonic Trap in the MCE Effect

**Figure 11:**
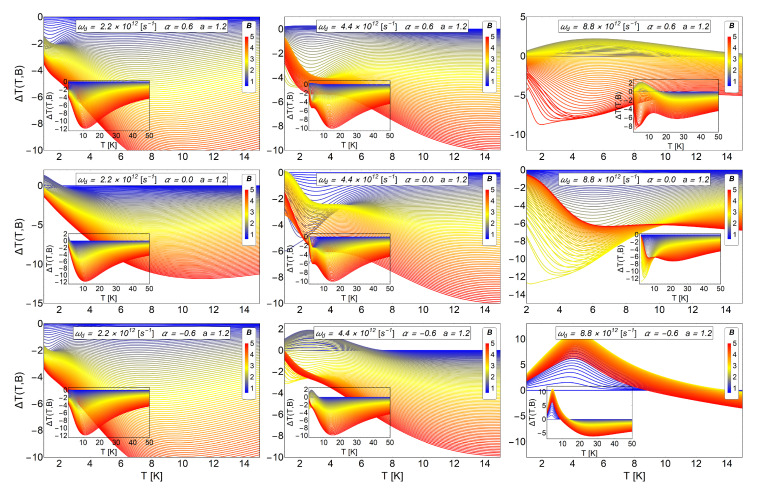
MCE effect (ΔT) for three different values of harmonic trap frequencies and three different values of AB-flux, with a fixed value of the antidot radius a=1.2. **Upper row:** We display the case of α=0.6, **middle row:**α=0 and **lower row:**α=−0.6. **Left column:** We treat the case of parabolic trap frequency $\omega_{d}=2.2\times 10^{12}$ s ${}^{-1}$, which in terms of energy represent 1.448 meV. **Central column**: The case of $\omega_{d}=4.4\times 10^{12}$ s${}^{-1}$, which in terms of energy represent 2.896 meV. **Right column:** The case of $\omega_{d}=8.8\times 10^{12}$ s${}^{-1}$, which in terms of energy represent 5.792 meV. The inset in each figure shows ΔT in a larger range of temperature, up to T=50 K. In general we observe an enhancement of the positive peak in the MCE for the system with higher frequency. In addition, the differences in the MCE for the cases with positive and negative AB fluxes can be noticed in the system with higher frequency. Therefore, there is a clear way to distinguish an AB flux by measuring the MCE. The quantity ΔT(T,B) is in units of Kelvin. Here, the horizontal axis represents the initial temperature of the system.

In the two previous subsections, it was shown that the antidot radius and the AB-flux could be used to control, in the low-temperature range, an oscillation in the MCE of direct-inverse type. In addition, we observe that an increase of the parameter α does not produce necessarily an increase in the positive peak in the MCE. In the case of the modification of the *a* parameter (antidot radius), the ΔT does not increase significantly for significant variations of the parameter, and even the oscillation in the MCE tends to disappear for a≥5. Therefore, to have control of the magnetocaloric response in the system, it is important to find a suitable parameter set, ($\omega_{d}$, *a* and α), that allows us to drive the MCE. Next, in Figure 11, we present results by varying the dot frequency, keeping fixed the antidot radius and for the three cases of AB fluxes, positive, negative and zero.

The dot frequency can be changed by modifying the parabolic trap on the sample. If we compare the cases shown in the lower row of Figure 11, we clearly appreciate an increase of the direct peak response in the MCE around T∼4.5 K. The value of ΔT is close to 11 K for a $B_{i}=0.6$ T and $B_{f}∼3$ T as we see in the last image of the lower row in Figure 11. The only parameter that has been changed corresponds to the frequency of the harmonic trap, increasing its value two times as compared with the value used in the central column. As we can see from the last figure of the central column, beyond to T∼12 K, the inverse MCE is recovered. Increasing the frequency of the harmonic trap causes the electron to be more confined near the center of the antidot. The confinement in the central area of the antidot makes the electron to stronger feel the AB-flux because the magnetic potential vector decays as 1/r away from the solenoid.

Therefore, this result allows to control the size of the magnetocaloric response (i.e., the ΔT peak) with the parameter $\omega_{d}$ of the present model. For the case of the quantum dot with spin, in reference [41], the oscillation of the MCE is destroyed for higher values of $\omega_{d}$ and only direct MCE is obtained. Here, we find that the peak of the direct MCE increases without suppressing the oscillations of the MCE. This is an advantage of antidot over the dot because we obtain a different type of magnetocaloric response, which can be used for adiabatic demagnetization refrigerators and magnetic field sensors [57].

However, for a more fundamental reason, we have demonstrated that under the controlled election of parameters (*a* and $\omega_{d}$), the AB flux can be determined by measuring MCE. i.e., the switching on or off of the AB flux can be detected by measuring the temperature difference that this switching provokes. This constitutes an alternative way to detect AB fluxes as compared to the standard effect of interference.

### 3.4. The Role of the Spin in the MCE Effect for Antidot

In order to complement the results presented in the previous subsections, we also take into account the electron spin of value $\frac{\hbar \hat{\sigma }}{2}$ and magnetic moment $\mu_{B}$, where $\hat{\sigma }$ is the Pauli spin operator and $\mu_{B}=\frac{e\hbar }{2m^{*}}$. Here the spin can have two possible orientations; one is ↑, and the other corresponds to ↓ with respect to the applied external magnetic field *B* in the direction of the z-axis. Therefore, we need to add the Zeeman term in the Bogachek-Landman energy levels presented in Equation (6). Consequently, the new energy spectrum is given by

(19) $$\begin{array}{c} E_{nm\sigma }^{ad}=\hbar \Omega \left(2n+{\left[{\left(m+\alpha \right)}^{2}+a^{2}\right]}^{1/2}+1\right)+\frac{1}{2}\hbar \omega_{c}\left(m+\alpha \right)-\mu_{B}\sigma B. \end{array}$$

The partition function can be easy calculated in the form

(20) $$\begin{array}{ccc} Z & = & \sum_{n}e^{-2\beta \hbar \Omega \left(n+\frac{1}{2}\right)}\sum_{m}e^{-\beta \hbar \Omega {\left[{\left(m+\alpha \right)}^{2}+a^{2}\right]}^{\frac{1}{2}}-\frac{\beta \hbar \omega_{B}}{2}\left(m+\alpha \right)}\sum_{\sigma }e^{\mu_{B}\sigma B}\\ & = & \mathrm{csch}\left(\beta \hbar \Omega \right)\cosh\left(\frac{\hbar \beta \omega_{B}}{2}\right)\sum_{m}e^{-\beta \hbar \Omega {\left[{\left(m+\alpha \right)}^{2}+a^{2}\right]}^{\frac{1}{2}}-\frac{\beta \hbar \omega_{B}}{2}\left(m+\alpha \right)} \end{array}$$

**Figure 12:**
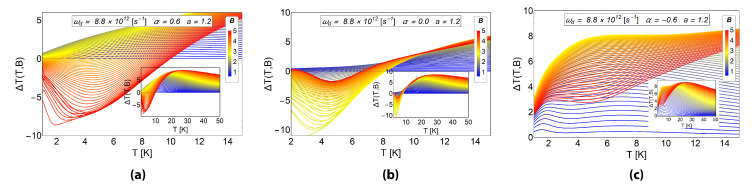
The MCE effect for a electron with spin in an antidot. For all graphics displayed in this figure, we use the value of $\omega_{d}=8.8\times 10^{12}$ s${}^{-1}$ and for *a* para meter the value of a=1.2. This case corresponds to the one shown in Figure 11, right column, for a spinless electron. For (**a**) we select α=0.6, for (**b**) the case of α=0 and for (**c**) α=−0.6. The quantity ΔT(T,B) is in units of Kelvin. Here, the horizontal axis represents the initial temperature of the system.

In Figure 12 we see the effect of the AB flux on the MCE as a function of temperature and magnetic field for the system with spin and fixed frequency of the dot ($\omega_{d}=8.8\times 10^{12}$ s${}^{-1}$), and fixed antidot radius, (a=1.2). These results have to be compared with Figure 11, right column, corresponding to the same set of parameters but for a spinless electron. We notice that the inclusion of the electron spin changes the overall behaviour of the MCE, however, noticeable differences are still present when comparing the cases of α>0, α<0 and α=0. At final external fields around and above B=4 T, and in the temperature range of 2 K <T<6 K, we have a large negative, ΔT<0, response for α=0.6, a small negative response for α=0, but a large positive effect, (ΔT>0) for α=−0.6. Therefore, in this region of parameters, we can design an experiment to detect the presence of a positive or negative AB-flux.

## 4. Conclusions

In this work, we explored the MCE effect for a parabolic trapped electron in an antidot, subjected to a uniform external field and under an Aharonov-Bohm flux. The model used is the one proposed by Bogachek and Landman model [52], that constitutes a combination of repulsive potential ($U\left(r\right)\propto r^{-2}$) and attractive potential ($U\left(r\right)\propto r^{2}$) leaving the electron confined in a ring shape finite region of space. We analysed all thermodynamics quantities and obtained the variation of the entropy and the temperature along the adiabatic strokes that characterize the MCE. In particular, we found a transition between the direct magnetocaloric response to inverse type by two different parameter changes: the antidot radius (a) and the AB-flux (α). We report that a small and big antidot radius only present inverse MCE effect. For values of the antidot radii between 1.5<a<5 we obtain a peak in the magnetocaloric response of direct type for low-temperature behaviour (less than 7 K). This ΔT is superior to 1 K for a small variation in the external magnetic field (close to 0.1 T). For the case of AB-flux, we note that for a small radius of the antidot structure, AB-flux generates a direct response on the MCE effect, when the parameter α reaches up to 0.5. For values higher than α=0.5, we note that the oscillation of direct-inverse type tends to disappear. Additionally, by reversing current in the solenoid (α<0), we found similar results to the previous case, but only for values of α greater than 0.5 in absolute value. Moreover, we show an advantageous form to increase the peak in the direct MCE without losing the oscillatory behaviour found for antidot radius and AB-flux, which is the manipulation of the frequency of the harmonic trap, that confines the electron more or less to a finite region of space.

Finally, we have demonstrated that under the controlled election of parameters, the switching on or off of an Aharonov-Bohm flux can be detected by measuring the magnetocaloric effect. The effect of the interactions among antidots in the MCE is currently under study. 

References
